# A genome-wide association, polygenic risk score and sex study on opioid use disorder treatment outcomes

**DOI:** 10.1038/s41598-023-49605-0

**Published:** 2023-12-15

**Authors:** Alannah McEvoy, Caroul Chawar, Amel Lamri, Jacqueline Hudson, Luciano Minuzzi, David C. Marsh, Lehana Thabane, Andrew D. Paterson, Zainab Samaan

**Affiliations:** 1 Department of Psychiatry and Behavioural Neurosciences, McMaster University, 100 West 5th St., Hamilton, ON L8N 3K7 Canada; 2 Department of Medicine, McMaster University, 1280 Main St. W., Hamilton, ON L8S 4L8 Canada; 3 NOSM University, 935 Ramsey Lake Rd., Sudbury, ON P3E 2C6 Canada; 4 Department of Health Research Method, Evidence & Impact, 1280 Main St. W., Hamilton, ON L8S 4L8 Canada; 5 Program in Genetics and Genome Biology, The Hospital for Sick Children, 686 Bay Street, Toronto, ON M5G 0A4 Canada; 6 Divisions of Biostatistics and Epidemiology, Dalla Lana School of Public Health, University of Toronto, 686 Bay Street, Toronto, ON M5G 0A4 Canada

**Keywords:** Genome-wide association studies, Addiction

## Abstract

Opioid use disorder continues to be a health concern with a high rate of opioid related deaths occurring worldwide. Medication Assisted Treatments (MAT) have been shown to reduce opioid withdrawal, cravings and opioid use, however variability exists in individual’s treatment outcomes. Sex-specific differences have been reported in opioid use patterns, polysubstance use and health and social functioning. Candidate gene studies investigating methadone dose as an outcome have identified several candidate genes and only five genome-wide associations studies have been conducted for MAT outcomes. This study aimed to identify genetic variants associated with MAT outcomes through genome-wide association study (GWAS) and test the association between genetic variants previously associated with methadone dose through a polygenic risk score (PRS). Study outcomes include: continued opioid use, relapse, methadone dose and opioid overdose. No genome-wide significance SNPs or sex-specific results were identified. The PRS identified statistically significant results (p < 0.05) for the outcome of methadone dose (R^2^ = 3.45 × 10^–3^). No other PRS was statistically significant. This study provides evidence for association between a PRS and methadone dose. More research on the PRS to increase the variance explained is needed before it can be used as a tool to help identify a suitable methadone dose within this population.

## Introduction

Opioid use disorder (OUD) continues to be a health concern with a high rate of opioid related deaths being reported worldwide with approximately 115,000 deaths from opioid overdose in 2017^1^. In Canada, 5368 apparent opioid related deaths occurred between January to September of 2021 with an increasing trend of opioid related deaths from 2016 to 2020^2^. OUD is a chronic relapsing condition and Medication Assisted Treatments (MATs) are critical parts of the strategy to address the opioid epidemic, and include the controlled administration of opioid agonists or antagonists^3,4^. The World Health Organization recommends both methadone and buprenorphine/naloxone (also known as suboxone) as MATs^1,3^.

Methadone Maintenance Treatment (MMT) has been reported to decrease opioid cravings and opioid use, with the treatment target aiming to help individuals control opioid use and regain stability^5–7^. While methadone can be effective, there is a large variability in the effective methadone dose, if individuals are on too low of a dose they may experience withdrawal symptoms and if too high of a dose, they may experience drowsiness, confusion and mental impairment^8^. Of further concern, inappropriate dosing of methadone may lead to relapse or increase the risk of overdose due to direct effects or interacting with other illicitly used opioids, and as such, continued opioid use is one of the most common risk factors for mortality among patients receiving MMT^9–11^. Further, while MMT has been shown effective in reducing rates of relapse, there are a number of individuals who continue to use illicit opioid while in treatment and are at risk of opioid overdose, with some reports of continued opioid use while on MMT being as high as 78%^12–14^. Thus, it is important to consider outcomes of MMT in addition to methadone dose such as continued opioid use, relapse and opioid overdose.

Further to individual differences, males and females are known to differ in addiction susceptibility and behaviour. Sex differences in opioid use patterns, polysubstance use and health and social functioning in patients receiving MAT have been previously reported^15–18^. In psychiatric disorders in which disparities in prevalence rates exist, genetic studies have identified higher burden of genetic risk in the less affected sex and different SNPs identified with a disorder in males and females, however no consistent patterns have emerged^19,20^. Thus, it is important to continue to investigate possible genetic differences by sex.

Due to the variability in individual methadone dose tolerance and risk, interest in a genetic predisposition to MMT outcomes has been the focus of research^21–25^. The most commonly studied Single Nucleotide Polymorphisms (SNPs) associated with MMT outcomes (including opioid addiction, methadone dose, methadone metabolism and plasma concentrations, opioid cessation, response to treatment and continued opioid use) are located within *OPRM1*, *OPRD1*, *ABCB1*, and *CYP2B6*^23,26,27^. However, many of the genetic studies assessing the pharmacogenetics of MAT are candidate gene studies with small sample size. A recent systematic review identified five genome-wide association studies (GWAS) investigating MAT outcomes, with only 3 loci reaching genome-wide significance^26^. The GWASs varied in outcomes as well as ethnicities with only two studies sharing an outcome of methadone dose^28,29^ and two studies with African American and European American participants^28,30^, two with Han Chinese participants^29,31^ and one with European participants^32^. The GWAS showed a lack of replication likely due to differences in outcomes and ethnicities, however one GWAS identified significant SNPs within *OPRM1*^26,28^.

GWAS are hypothesis free and can identify a large number of genetic variants associated with a trait. However, with growing evidence suggesting that the majority of genetic variants have small effects which collectively contribute to the risk of a disease, it is important to consider other methods in addition to GWASs, such as a Polygenic Risk Score (PRS). PRSs are able to aggregate the effects of variants across the genome through creating a weighted sum of the number of risk alleles an individual carries based on the assumption of a previously conducted GWAS^33–35^.

With the few GWAS significant loci for MAT outcomes and the risk of mortality faced by this population, we aimed to identify genetic variants associated with MMT outcomes, including continued opioid use, relapse, methadone dose and opioid overdose via GWAS. Further, we aimed to determine the combined effect of known genetic variants associated with methadone dose in the literature associated with MMT outcomes in our population through a Polygenic Risk Score (PRS).

## Objectives

This study aimed to identify novel genetic variants and test the association between genetic variants previously associated with MMT outcomes in a clinical sample. The study objectives are to:

1. Conduct a GWAS to identify genetic variants with various MMT outcomes.
2. Investigate sex differences of any genetic variants approaching or reaching genome-wide significance.
3. Conduct a PRS based on a large previously published GWAS on MMT.

## Methods

In accordance with the Strengthening the Reporting of Genetic Association studies (STREGA) guidelines, an extension of Strengthening the Reporting of Observational studies in Epidemiology (STROBE) statement, an accompanying STREGA checklist can be found in Supplementary File 1^36^.

### Study design and setting

Data were collected as part of the GENetics of Opioid Addiction (GENOA) and Pharmacogenetics of Opioid Substitution Treatment Response (POST) programs. The GENOA and POST studies are prospective cohort studies conducted in collaboration with the Canadian Addiction Treatment Centre (CATC) and McMaster University. The GENOA and POST studies were designed to identify factors associated with opioid use and treatment outcomes including genetic risk factors in patients diagnosed with OUD and receiving treatment^37^. Details on the GENOA and POST studies have been previously reported^37^. Briefly, participants (n_GENOA_ = 1536, n_POST_ = 3319) were recruited from 76 CATC sites across Ontario, Canada (GENOA, 2013–2016, POST, 2018–present). Participants from the POST study included in this analysis were recruited in 2018–2019. At study recruitment, participants completed an extensive interview with a trained researcher and were asked to provide a DNA sample. Participants in the GENOA study provided a blood sample for DNA and participants in the POST study provided a saliva sample for DNA. As previously reported, participants were followed for a 12-month period through their electronic medical record which documented their weekly or biweekly Urine Drug Screens (UDS). At study recruitment, participants UDS for 3-months prior to study entry was collected, thus a total of 15-months of UDS were obtained. Participants were given a coffee shop gift card of low monetary value after every face-to-face interview in appreciation for participating^17,38^.

### Participants

While GENOA and POST studies had similar methods, differences in inclusion criteria exist. For the GENOA study, patients were eligible to participate if they were 18 years or older and met the criteria for Diagnostic and Statistical Manual—fourth edition (DSM-IV) opioid addiction requiring treatment (later replaced in Diagnostic and Statistical Manual—fifth edition (DSM-5) as Opioid Use Disorder (OUD))^39^. For the POST study, patients were eligible to participate if they were 16 years or older and met the criteria for DSM-5 opioid use disorder (OUD). For both the GENOA and POST study, patients were excluded if they did not speak English or refused to provide a blood (GENOA) or saliva (POST) sample for DNA.

### Eligibility criteria

In addition to meeting eligibility for the GENOA or POST study criteria, further inclusion criteria for this study included being on MMT. For the measures of continued opioid use and relapse participants had to have UDSs assessing for the presence of opioids for a minimum duration of 3 months and 6 months, respectively. For continued opioid use and relapse, participants were excluded from the analysis if they self-reported a current prescription for opioids due to the uncertainty of whether UDS for opioids, which did not include UDS screens for methadone, would be from licit or illicit use. For the measures of methadone dose and opioid overdose participants were excluded if they did not self-report methadone dose or if they did not answer the self-report question on lifetime opioid overdoses, respectively. Participants in the GENOA study were not asked about lifetime opioid overdoes, and were therefore excluded from the opioid overdose analyses.

### Variables and quantitative variables

Outcomes measured in the study include the following:

1. Continued opioid use; defined as any opioid positive UDS observed over a duration of 3 to 15 months, measured as a binary variable.
2. Relapse; defined as an event of an opioid positive UDS following at least 3 months of opioid negative UDSs, measured as a binary variable.
3. Methadone dose; defined as the daily amount of methadone a patient is administered at the time of study recruitment in milligrams, measured as a continuous variable.
4. Opioid overdose; defined as any self-reported opioid overdose reported in their lifetime, measured as a binary variable.

Covariates for the measures of continued opioid use, relapse and opioid overdose that were accounted for in the statistical models included: sex, age in years, dose of methadone in milligrams, duration on MMT in months and three genetic principal components accounting for differences due to population stratification. Covariate for the measure of methadone dose that were accounted for in the statistical models included: sex, age in years, duration on MMT in months, weight in kilograms and three genetic principal components accounting for differences due to population stratification. To create the principal components, a principal component analysis was conducted with the data using PLINK prior to data imputation. Further details on the principal component analysis have been previously reported^37^.

### Data sources/measurement

Urine drug screens were completed on average once a week. Urine samples were tested, analyzed and reported as the number of positive screens for the drug detected in the test using the FaStep Assay (Trimedic Supply Network Ltd, Concord, Ontario, Canada)^40^. Methadone dose and opioid overdose was self-reported by participants at study recruitment. Methadone dose was transformed by dividing by 10 for clinical interpretation, as changes in 10 mg of methadone was deemed as clinically meaningful compared to changes in 1 mg of methadone and using the log to approach a normal distribution.

### Quality control checks

As part of the GENOA study, whole blood samples were collected for DNA. Blood samples were centrifuged, separated and frozen in − 20 °C within 2 h of collection at the clinics and then transferred to − 80 °C freezers located at McMaster University within 1 month of collection. As part of the POST study, approximately 2 ml of saliva samples were collected at the baseline using DNAgenotek all-in-one system for the collection, stabilization and transportation of DNA from saliva (OGR-500)^41^. DNA was extracted from blood or saliva samples^42^ and genotyped by Genomé Quebec using GenomeStudio (v 2.0.4) and the Infinium Global Screening Array—24 v1.0^43–45^. R version 3.3.3 was used for quality control checks and variant quality control procedures were applied using PLINK v1.90^46,47^.

Samples were excluded if they have a low variant call rate (< 99%), inconsistencies between self-reported vs. genetically determined sex or ancestry or if they exhibited excess heterozygosity suggestive of sample contamination (exchange of DNA between two or more samples). An identity-by-state/decent computation was performed to identify and exclude duplicates as well as first and second-degree relatives. Variants were excluded if they have a low call rate across samples (< 99%), or if the minor allele frequency was less than 0.05. As participants were predominantly European descent (80.7%), only those who were of European descent were selected for imputation. Imputation was completed by TOPMed Imputation Server (Version R2) using the software Eagle v2.4 and Minimac4 for phasing^48–51^. Post-imputation filtering excluded SNPs with Rsq quality metrics of less than 0.3 and minor allele frequencies lower than 0.05. Further information on the quality control steps were previously reported^37^.

### Bias

Although measures were taken to identify and mitigate areas of bias, potential sources of bias remained. While outcomes of continued opioid use and relapse were defined through UDSs to provide an objective measure, methadone dose and opioid overdose were self-reported, allowing for potential social desirability or recall biases. Sex-differences have been identified in social desirability biases, such that differing response may have occurred between males and females based on what response seemed more desirable^52^. Further, it is possible that participants did not accurately recall what their current methadone dose is or if they have experienced an opioid overdose. Additionally, the current study may be biased by volunteer bias. In addition to bias that exists within individuals for the participation of research, biases exist in participants who are willing to participate in genetic studies, thus the sample population may not be representative of the entire OUD population receiving treatment^53^. Lastly, due to the observational nature of this study, it is not possible to control for all extraneous confounding variables that may exist.

### Study size

Of the 4621 participants with genetic samples available, we excluded 775 samples from a cohort not eligible to be included due to different measures and we excluded participants based on ancestry as we did not have adequate power to perform subgroup analysis. In total, 2251 participants of European ancestry passed the genetic quality control steps and were used for this study.

### Statistical measures

Descriptive statistics were reported on the total sample, by sex, to describe the demographic and clinical characteristics. Continuous variables were expressed as means with standard deviations, while categorical variables were expressed as counts.

Separate regression analyses were preformed to test the association between the outcomes and genetic variants. Logistic regressions were conducted for the outcomes of continued opioid use, relapse, and opioid overdose and a linear regression was used for the outcome of methadone dose. An additive model was used to test the association of each genetic variant and the phenotype of interest. All aforementioned covariates were adjusted for in their respective analyses. Identical regression analyses as above were conducted separately for male and female subsets, as well as a separate model including an interaction between SNP and sex, for SNPs approaching, or meeting, genome-wide significance.

For the PRS, summary statistics of SNPs from a GWAS, chosen for its outcome, investigating daily methadone dose, and using only the European ancestry summary statistics to match our ancestry population^28^. The genomic position of the selected variants were converted from GRCh37 to GRCh38 to match our data using the UCSC*liftOver* tool^54^. The GWAS summary statistics results were pruned using PRSice-2 whereby sites within 250 kb of each index variant and with r^2^ > 0.5 were pruned out. Subset of SNPs were selected at p-value thresholds of 0.0001 and 1 × 10^–5^, based on the previously reported GWAS data availability. PRSs were calculated separately for each outcome (continued opioid use, relapse, methadone dose, and opioid overdose), with all aforementioned covariates adjusted for in their respective analyses. The Bonferroni corrected p-value is < 0.025, as two PRSs were calculated for each outcome.

Samples with missing outcome values were excluded from the analysis. Missing values for the covariates of each analysis were imputed via mean substitution, from the averages of the values calculated per analysis using R studio 3.3.3^47,55^. For the outcome of continued opioid use, methadone dose and duration on MMT were imputed, and for the outcome of methadone dose, duration on MMT and weight were imputed using mean substitution.

All statistical analyses were performed on PLINK v1.09, R studio 3.3.3 and PRSice-2 (v 2.3.5)^46,47,55–57^. Regional plots of GWAS results were generated by LocusZoom^58^.

### Ethics approval and consent to participate

The GENOA and POST studies were performed in accordance with the Declaration of Helsinki and were reviewed and approved by the Hamilton Integrated Research Ethics Board (GENOA: 11-056, POST: 4556). Written informed consent was obtained from all participants.

## Results

### Participants

Of the 2251 participants included in this study, 2 were missing methadone dose amount and 94 had no UDS reported. Of the remaining 2157 participants with UDS available, 169 only had one time-point and were excluded from the relapse outcome. Finally, only 1327 of 2251 participants reported if they had previously experienced an opioid overdose. Therefore, 2157 were included in the continued opioid use analysis, 1988 in the relapse analysis, 2249 in the methadone dose analysis and 1327 in the opioid overdose analysis.

### Descriptive data

The current study included 924 participants from the GENOA study and 1327 from the POST study (Table 1). The majority of study participants were male (57.53%) and mean age was 39.3 (± 11.1 SD) years. It was most common for participants to report to never been married, unemployed and have less than a grade 12 education. Participants reported an average methadone dose of 71.8 mg/day and have been on treatment for 53.5 months, or approximately 4.5 years, and 2.36% reported currently having a prescription for opioids, other than methadone for medical indications, namely, pain conditions. Participants included in the current study who were recruited for the GENOA and POST study did not differ based on average age (GENOA = 38 years old, POST = 40 years old), sex (GENOA = 58% males, POST = 57% males), average methadone dose (GENOA = 73 mg/day, POST = 71 mg/day), or rate of continued opioid use (GENOA = 80%, POST = 79%) but did have different relapse rates (GENOA = 40%, POST = 27%). Finally, 6,377,206 SNPs passed the quality control steps and were included in the GWAS and of the 293 SNPs available from the previously reported GWAS summary statistics, 39 SNPs were included in the PRSs following pruning. The full list of participants demographics can be found in Table 1.

**Table 1:** Demographics.

	Total	Male	Female
N (%)	2251	1295 (57.53)	956 (42.47)
GENOA	924	539 (58.33)	385 (41.67)
POST	1327	756 (56.97)	571 (43.03)
Age in years, mean (SD)	39.26 (11.11)	40.06 (11.17)	38.19 (10.95)
Marital status, N (%)^a^			
Never married	1030 (48.75)	623 (51.70)	407 (44.82)
Common law	425 (20.11)	222 (18.42)	203 (22.36)
Currently married	194 (9.18)	111 (9.21)	83 (9.14)
Separated	202 (9.56)	99 (8.22)	103 (11.34)
Divorced	195 (9.23)	120 (9.96)	75 (8.26)
Widowed	67 (3.17)	30 (2.59)	37 (4.07)
Currently employed, N (%)^b^	717 (33.93)	487 (40.38)	230 (25.36)
Education, N (%)^c^			
Less than grade 9	516 (24.47)	296 (24.56)	220 (24.34)
Grade 9–12	974 (46.18)	606 (50.29)	368 (40.71)
Trade school	66 (3.13)	48 (3.98)	18 (1.99)
College/University/Graduate School	553 (26.22)	255 (21.16)	298 (32.96)
Methadone dose in mg/day, mean (SD)^d^	71.76 (43.07)	74.18 (44.57)	68.47 (40.75)
Duration on MMT in months, mean (SD)^e^	53.53 (58.92)	53.50 (56.89)	53.57 (61.61)
Current opioid prescription, N (%)^f^	53 (2.36)	31 (2.39)	22 (2.30)
Continued opioid use, N (%)^g^	1716 (79.55)	993 (80.02)	723 (78.93)
Relapse, N (%)^h^	631 (31.74)	362 (31.81)	269 (31.65)
Reported opioid overdose in lifetime, N (%)^i^	437 (32.93)	244 (32.28)	193 (33.80)

^a^Data available for n_Total_ = 2113, n_Male_ = 1205, n_Female_ = 908.

^b^Data available for n_Total_ = 2113, n_Male_ = 1206, n_Female_ = 907.

^c^Data available for n_Total_ = 2109, n_Male_ = 1205, n_Female_ = 904.

^d^Data available for n_Total_ = 2249, n_Male_ = 1294, n_Female_ = 955.

^e^Data available for n_Total_ = 2242, n_Male_ = 1290, n_Female_ = 952.

^f^Data available for n_Total_ = 2250, n_Male_ = 1295, n_Female_ = 955.

^g^Data available for n_Total_ = 2157, n_Male_ = 1241, n_Female_ = 916.

^h^Data available for n_Total_ = 1988, n_Male_ = 1138, n_Female_ = 850.

^i^Data available for n_Total_ = 1327, n_Male_ = 756, n_Female_ = 571.

### Main results

The lambda GC was within an acceptable range for all GWAS outcomes (0.99 for continued opioid use, 1.01 for relapse, 1.00 for dose and 1.01 for opioid overdose) (Supplementary Figs. 1, 3, 5, 7). We identified one variant approaching genome-wide significance for each outcome; for continued opioid use chr5:71,874,588:GT:G (odds ratio (OR) 1.62, 95% confidence interval (CI) 1.34, 1.97, p = 8.87 × 10^–7^), for relapse rs10912116 on chromosome 1 (OR 0.68, CI 059, 0.79, p = 4.40 × 10^–7^), for methadone dose rs6670338 on chromosome 1 (Beta = 0.04, Standard Error (SE) = 0.01, p = 5.79 × 10^–7^), and for opioid overdose rs12777585 on chromosome 10 (OR 1.55, CI 1.29, 1.86, p = 2.60 × 10^–6^). For all outcomes, using the top SNP as a covariate in the analysis did not result in any further significant results, thus no other strong signals within the region exist. Results for the lead SNPs for each respective outcome can be found in Table 1 in Supplementary File 2. Manhattan plots, QQ plots and regional plots for the respective study outcomes can be found in Figs. 1–12 in Supplementary File 2. Associations from the literature compared to our results can be found in Supplementary File 3, however our GWAS did not replicate any know genetic associations of MMT outcomes from the literature.

Results from the sex-stratified association analyses between the SNP approaching GWAS significance for each respective outcome are reported in Table 2 in Supplementary File 2, including separate models for males, females and the interaction, using the p-value of less than 0.05 to indicate statistical significance. The GT allele of 5:71,874,588:GT:G was significantly associated with an increased odds of continued opioid use in both males [OR 1.56, CI 1.19, 2.03, p = 1.09 × 10^–3^] and females [OR 1.70, 95% CI 1.28, 2.25, p = 2.65 × 10^–4^], and the sex by SNP interaction was not significant (p = 0.63). The T allele of rs10912116 was significantly associated with a decreased odds of relapse in both males [OR 0.77, 95% CI 0.63, 0.94, p = 8.87 × 10^–3^] and females [OR 0.59, 95% CI 0.47, 0.74, p = 5.07 × 10^–6^] and the sex by SNP interaction was nominally significant (p = 4.89 × 10^–2^). The A allele of rs6670338 was significantly associated with methadone dose in both males [Beta = 0.04, SE = 0.01, p = 8.21 × 10^–4^] and females [Beta = 0.05, SE = 0.01, p = 2.32 × 10^–4^], however, the sex by SNP interaction was not significant (p = 0.39). Finally, the G allele of rs12777585 was significantly associated with an increased odds of opioid overdose in both males [OR 1.67, 95% CI 1.31, 2.13, p = 3.76 × 10^–5^] and females [OR 1.40, 95% CI 1.06, 1.86, p = 1.88 × 10^–2^], however the sex by SNP interaction was not significant (p = 0.39).

The bar plots depicting the model of fit of the different PRSs across p-value thresholds for each outcome can be found in Figs. 13–16 in Supplementary File 2. Results from the PRS best-fit model for continued opioid use, relapse, methadone dose and opioid overdose are reported in Table 2, and interpreted with the significance threshold of p < 0.05. Using the p-value threshold of 1.0 × 10^–5^ for the previous GWAS summary statistics, the PRS was significantly associated with methadone dose (PRS R^2^ = 3.45 × 10^–3^, p = 5.42 × 10^–3^). The PRS assessed at the two p value thresholds were not significantly associated with other outcomes, namely continued opioid use, relapse and opioid overdose.

**Table 2:** PRS best model fit for each outcome.

Outcome	Threshold	PRS R^2^	Full R^2^	Null R^2^	Coefficient	SE	P	Number of SNPs
Continued opioid use	1.0 × 10^–5^	7.37 × 10^–4^	3.39 × 10^–2^	3.31 × 10^–2^	2.07	2.07	0.32	8
Relapse	0.0001	6.70 × 10^–4^	1.41 × 10^–2^	1.35 × 10^–2^	− 4.27	4.38	0.33	39
Methadone dose	1.0 × 10^–5^	3.45 × 10^–3^	5.20 × 10^–2^	5.43 × 10^–2^	− 0.54	0.20	**5.42 × 10**^**–3**^	8
Opioid overdose	0.0001	9.58 × 10^–4^	2.43 × 10^–2^	2.34 × 10^–2^	5.12	5.38	0.34	39

*PRS R*^*2*^ variance explained by the PRS, *Full R*^*2*^ variance explained by the full model, *Null R*^*2*^ variance explained by the covariates, *SE* standard error, *Number of SNPs* number of SNPs included in the model. Significant values are in bold.

## Discussion

The GWASs did not replicate any known genetic associations of MMT outcomes from the literature. Two SNPs, chr5:71,874,588:GT:G and rs10912116 on chromosome 1, have no known associations with other traits or pathways in the literature by a search of NHGRI-EBI GWAS catalog (July 2022) and are not found within a gene region^59,60^. The SNP associated with methadone dose, rs6670338, is in a intron of transforming growth factor beta receptor 3 (*TGFBR3*), however traits previously associated with SNPs in this gene include systolic blood pressure, ischemic stroke, diabetes and other traits that are not related to mental health conditions or addiction^59^. The SNP associated with opioid overdose, rs12777585, is in an intron of the heat shock protein family A member 12A (*HSPA12A*), wherein SNPs in this region have been previously associated with externalizing behaviours (namely attention deficit hyperactivity disorder, substance use, and antisocial behaviours), educational attainment and smoking initiation^59,61–63^. However, rs12777585 is not strong LD with any SNPs previously associated with the aforementioned traits (see Fig. 17 in Supplementary File 2 for an LD matrix). As OUD, or more specifically substance use behaviour as a whole, is classified as externalizing, as well as having phenotypic associations with education attainment and smoking behaviour, it is possible that this gene region could indicate these traits are not independent or reflect genuine pleiotropy^61,64–66^. Further investigation into *HSPA12A* is required to determine the genetic association with opioid overdose.

We found a statistically significant association of a PRS for methadone dose, suggesting it can be applied to assess the individual level variability in methadone dose. However, the variability explained by this PRS was small, 3.45 × 10^–3^, suggesting that much of the variance due to variants not captured by our PRS (variants not included in the GWAS summary statistics, low MAF, etc.) to other non-genetic (environment), or gene environment interactions. The PRSs for continued opioid use, relapse and opioid overdose were not significant and, similar to the PRS for methadone dose, the variability explained by genetics was minor (less than 6.70 × 10^–4^). It is important to note that the GWAS summary statistics from the literature investigated methadone dose, and thus SNPs contributing to individual variability in methadone dose may not contribute to genetic variability in the outcomes of continued opioid use, relapse and opioid overdose despite their clinical associations^28^. Thus, it is also important to consider that the lack of significant findings within this study may be due to shared genetic contribution of various substances of abuse or externalizing behaviours^61,67^.

Lastly, it is important to discuss the results of the sex-specific analyses as it has been previously reported that females are more likely to present to treatment with higher rates of psychiatric comorbidities, greater life instability, have higher relapse rates, and experience faster dependence progression rates and males are more likely to present to treatment with ongoing drug use and other risky drug-related behaviours than females^15,18,25^. Further, biological sex differences, including neuroanatomy and neurochemistry, as well as psychological and behavioural differences such as in cognition, aggression and neurological diseases^68^. Thus, while this study did not find sex-specific differences within MMT outcomes, it is important to continue to investigate the potential genetic differences that may exist in sex-specific treatment outcomes.

In addition to the sources of bias discussed earlier, limitations exist within the study. First, the results from this study are limited to those of European ancestry and therefore may not be generalizable to individuals from different ancestry backgrounds. Further, it is important to note the level of missing data. Due to the specific criteria of each outcome, multiple participants were excluded either due to a lack of UDS or, for opioid overdose specifically, not being a part of the POST study where data collection slightly varied from the GENOA study. It is important to mention that some participants with missing UDS could have left treatment due to a relapse (and continued opioid use), transferred to another clinic and reminded stable on MMT or entered another treatment facility. More importantly, it is important to note that we do not know their true outcome. Finally, it is important to note that methadone dose or UDS may not have been an accurate measure of treatment response as treatment outcomes can be complex and no one definition has been agreed upon to be the ultimate treatment response^69^. In addition, study participants were enrolled in different stages of treatment, as such participants could have been at induction, treatment stabilization or taper stages, each of which vary the amount of methadone as well likelihood of opioid use based on opioid cravings^70^. While we did control for duration of treatment, our definition was that if a participant restarted treatment within a year, the participant would count that as their current treatment (e.g. if a participant had restarted treatment 5 times over the course of 2 years, 24 months was entered as duration on treatment). Therefore, participants opioid use, relapse rate, methadone dose and risk of overdose may have been impacted by their stage of treatment despite efforts to control for this in our analyses.

## Conclusion

This study provides additional insight into the genetics of MMT response. While further research is required to understand the complexity of OUD and treatment outcomes, it is important to continue to investigate genetic differences in MMT response given the known individual level variability and future clinical implications of personalized care.

### Supplementary Information

Supplementary Information 1.

Supplementary Information 2.

Supplementary Information 3.

## Data Availability

Summary statistics are available to download from Locus Zoom. Continued Opioids Use: https://my.locuszoom.org/gwas/181183/?token=177361adf2de4d8bb0ac9410ed6e1eb9. Relapse: https://my.locuszoom.org/gwas/232189/?token=95c59906baa844e2933a0950c0600771. Methadone Dose: https://my.locuszoom.org/gwas/715902/?token=82c590b81ad442f6a912ff9fb3a23d38. Opioid Overdose: https://my.locuszoom.org/gwas/524614/?token=23054b424e1c4e0eb3b8dfd8cb3718dc.

## Abbreviations

CATC: Canadian Addiction Treatment Centre
CI: Confidence interval
DSM-IV: Diagnostic and Statistical Manual—fourth edition
DSM-5: Diagnostic and Statistical Manual—fifth edition
GENOA: GENetics of Opioid Addiction
GWAS: Genome-Wide Association Study
MAT: Medication Assisted Treatment
MMT: Methadone Maintenance Treatment
OR: Odds ratio
OUD: Opioid use disorder
POST: Pharmacogenetics of Opioid Substitution Treatment Response
PRS: Polygenic risk score
SNP: Single nucleotide polymorphism
SE: Standard error
STREGA: Strengthening the Reporting of Genetic Association studies
STROBE: Strengthening the Reporting of Observational studies in Epidemiology
UDS: Urine drug screens
