# Supplementary material for: A genome-wide association, polygenic risk score and sex study on opioid use disorder treatment outcomes
Source: Sci Rep. 2023 Dec 15;13:22360. doi: 10.1038/s41598-023-49605-0 (PMC10724251; doi:10.1038/s41598-023-49605-0)
Supplement: Supplementary file 2 — Supplementary Information 2. [file 41598_2023_49605_MOESM2_ESM.docx]

A genome-wide association, polygenic risk score and sex study on Opioid use disorder treatment outcomes

Supplementary File 2

| Supplementary Table 1. Lead SNPs from each GWAS | | | | | | | | | |
| --- | --- | --- | --- | --- | --- | --- | --- | --- | --- |
| **Outcome** | **Chr** | **SNP** | **BP (GRCh 38)** | **A1** | **A2** | **MAF** | **OR/BETA*** | **95% CI/SE*** | **P** |
| **Continued Opioid Use** | 5 | rs5868616 | 71874588 | GT | G | 0.236 | 1.62 | 1.34,1.97 | 8.87×10^-7^ |
| **Relapse** | 1 | rs10912116 | 187657898 | T | C | 0.348 | 0.68 | 0.59, 0.79 | 4.40x10^-7^ |
| **Methadone Dose** | 1 | rs6670338 | 91764748 | G | A | 0.237 | 0.04 | 0.01 | 5.79x10^-7^ |
| **Opioid Overdose** | 10 | rs12777585 | 116685252 | C | G | 0.293 | 1.55 | 1.29, 1.86 | 2.60x10^-6^ |
| Model adjusted for age, sex, dose of methadone (mg), duration on methadone in months and principal components for continued opioid use, relapse and opioid overdose. Model adjusted for age, sex, duration on methadone in months, weight in kilograms and principal components for methadone dose. *Odds ratio and confidence interval reported for binary variables and Beta and standard error for continuous variables.  Chr=chromosome, SNP=single nucleotide polymorphism, BP=base pair, A1=reference allele, A2=alternative allele, MAF=minor allele frequency (reference allele), OR=odds ratio of A1, BETA= beta coefficient, 95 % CI = 95% Confidence Interval, SE = Standard Error | | | | | | | | | |

| Supplementary Table 2. SNPs and associated outcomes stratified by sex | | | | | | | |
| --- | --- | --- | --- | --- | --- | --- | --- |
| **Outcome** | **SNP** | **N** | **Reference Allele** | **MAF** | **OR/BETA*** | **95 % CI/SE*** | **P** |
| **Continued Opioid Use** | **rs5868616** |  | GT |  |  |  |  |
|  | Males | 1241 |  | 0.222 | 1.56 | 1.19, 2.03 | **1.09x10^-3^** |
|  | Females | 916 |  | 0.253 | 1.70 | 1.28, 2.25 | **2.65x10^-4^** |
|  | Interaction | 2157 |  | 0.236 | 1.10 | 0.75, 1.62 | 0.63 |
| **Relapse** | **rs10912116** |  | T |  |  |  |  |
|  | Males | 1138 |  | 0.345 | 0.77 | 0.63, 0.94 | **8.87x10^-3^** |
|  | Females | 850 |  | 0.361 | 0.59 | 0.47, 0.74 | **5.07x10^-6^** |
|  | Interaction | 1988 |  | 0.352 | 0.74 | 0.55, 1.00 | **4.89x10^-2^** |
| **Methadone dose** | **rs6670338** |  | A |  |  |  |  |
|  | Males | 1294 |  | 0.239 | 0.04 | 0.01 | **8.21x10^-4^** |
|  | Females | 955 |  | 0.235 | 0.05 | 0.01 | **2.32x10^-4^** |
|  | Interaction | 2249 |  | 0.237 | 0.01 | 0.02 | 0.39 |
| **Opioid Overdose** | **rs12777585** |  | G |  |  |  |  |
|  | Males | 756 |  | 0.296 | 1.67 | 1.31, 2.13 | **3.76x10^-5^** |
|  | Females | 571 |  | 0.289 | 1.40 | 1.06, 1.86 | **1.88x10^-2^** |
|  | Interaction | 1327 |  | 0.293 | 0.85 | 0.59, 1.23 | 0.39 |
| Model adjusted for age, dose of methadone (mg), duration on methadone in months and principal components for continued opioid use, relapse and opioid overdose. Model adjusted for age, duration on methadone in months, weight in kilograms and principal components for methadone dose. *Odds ratio and confidence interval reported for binary variables and Beta and standard error for continuous variables.  N=number of participants included in analysis, MAF=minor allele frequency, OR=odds ratio, BETA= beta coefficient, 95% CI = 95% confidence interval levels (lower, upper), SE=standard error | | | | | | | |

Supplementary Figure 1. Continued opioid use Manhattan Plot (n=2,157)


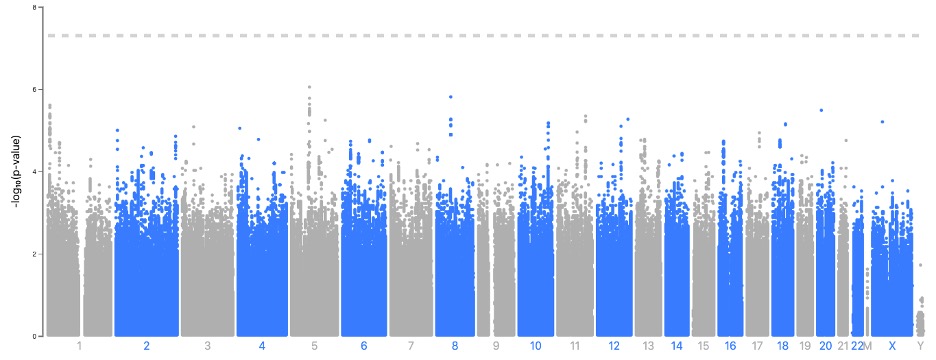


Supplementary Figure 2. Continued opioid use QQ Plot


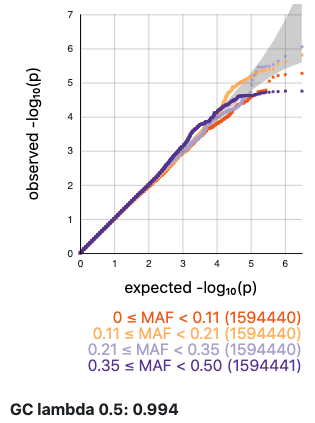


Supplementary Figure 3. Reginal plot for chr5:71874588:GT:G approaching genome-wide significance for continued opioid use from LocusZoom[1] using European for LD information


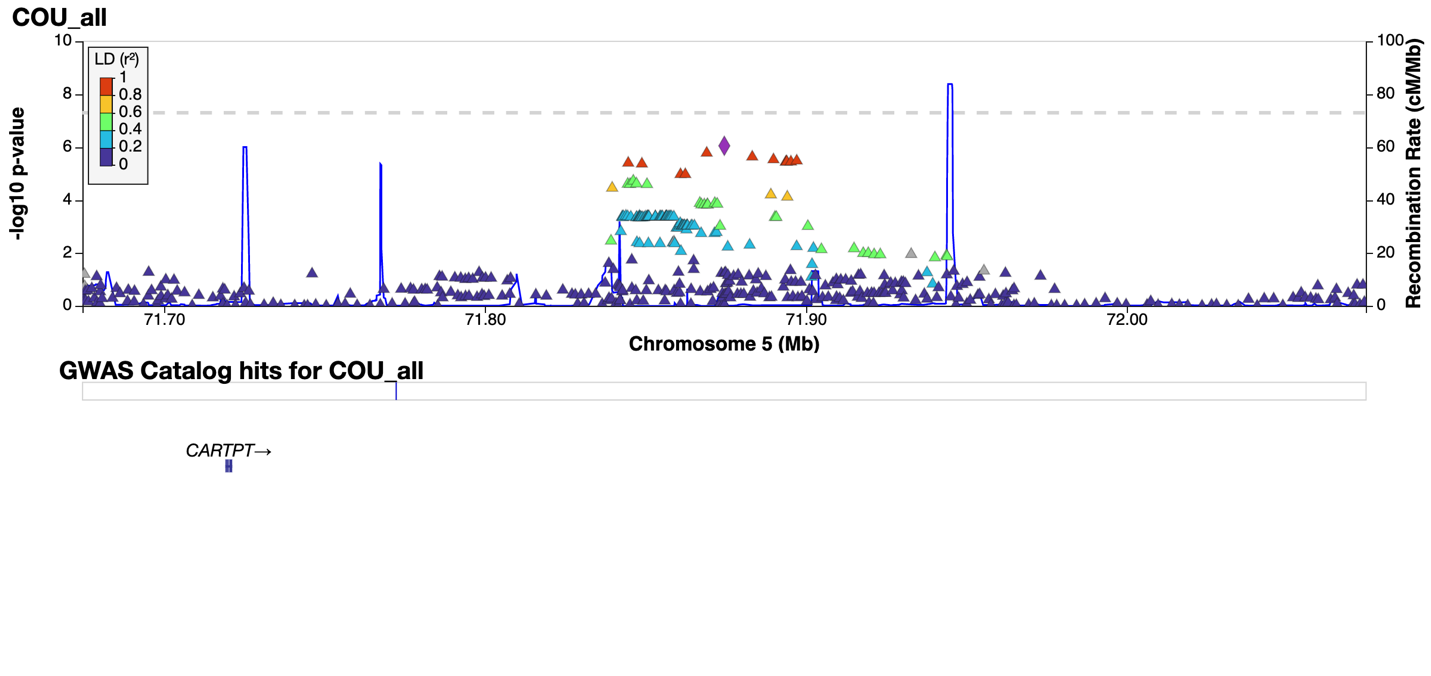


Supplementary Figure 4. Relapse Manhattan Plot (n=1,988)


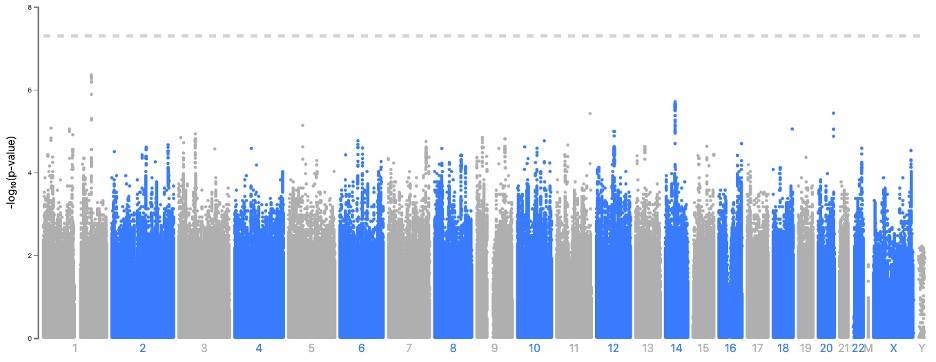


Supplementary Figure 5. Relapse QQ Plot


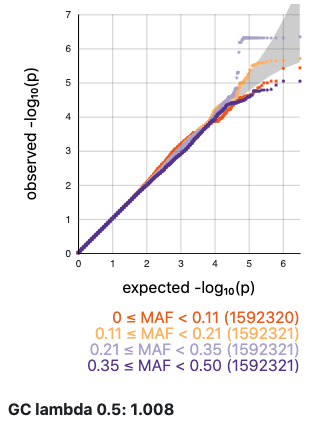


Supplementary Figure 6. Reginal plot for rs10912116 approaching genome-wide significance for relapse from LocusZoom[1] using European for LD information


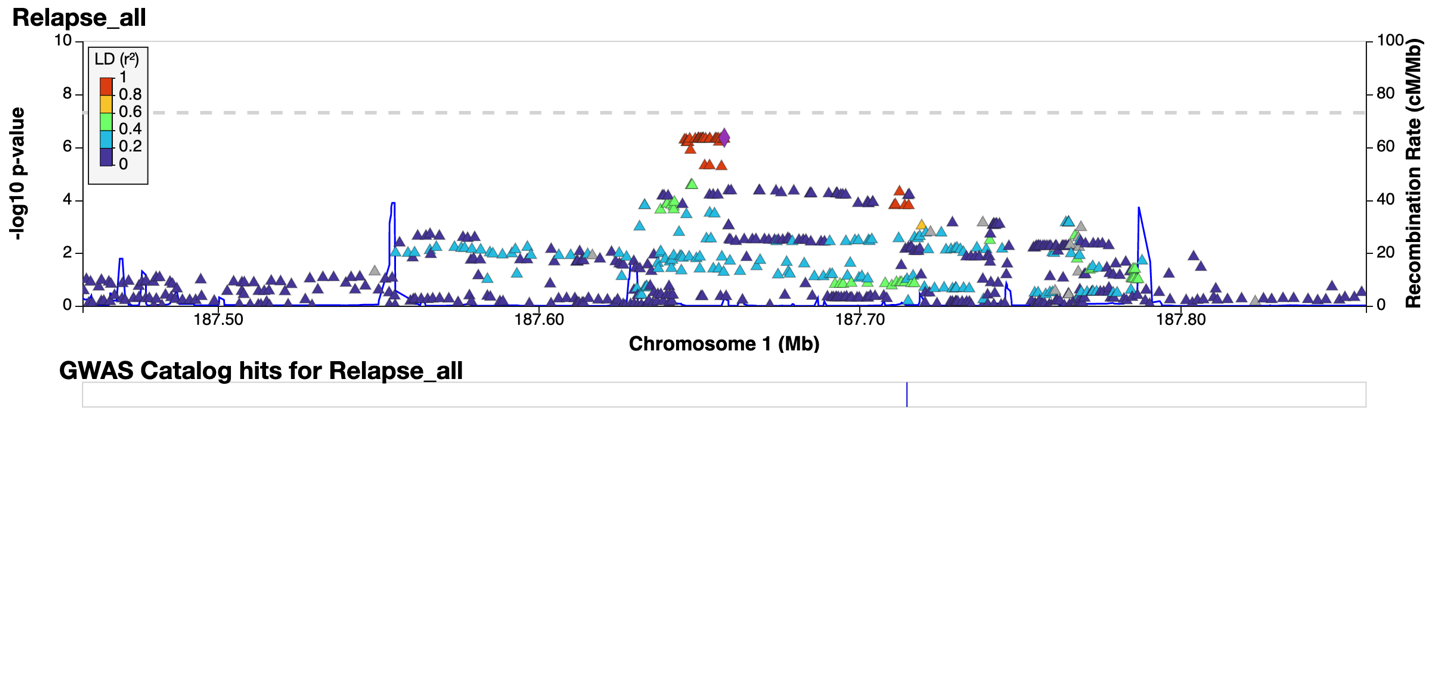


Supplementary Figure 7. Methadone dose Manhattan Plot (n=2,249)


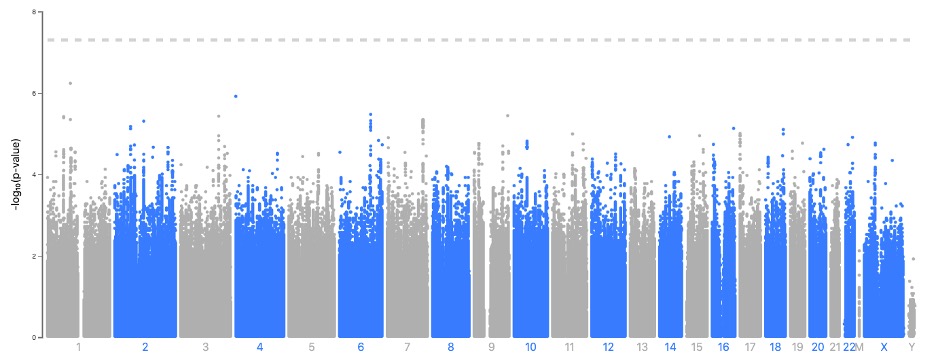


Supplementary Figure 8. Methadone dose QQ Plot


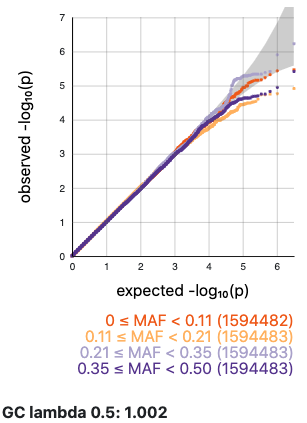


Supplementary Figure 9. Reginal plot for rs6670338 approaching genome-wide significance for methadone dose from LocusZoom[1] using European for LD information


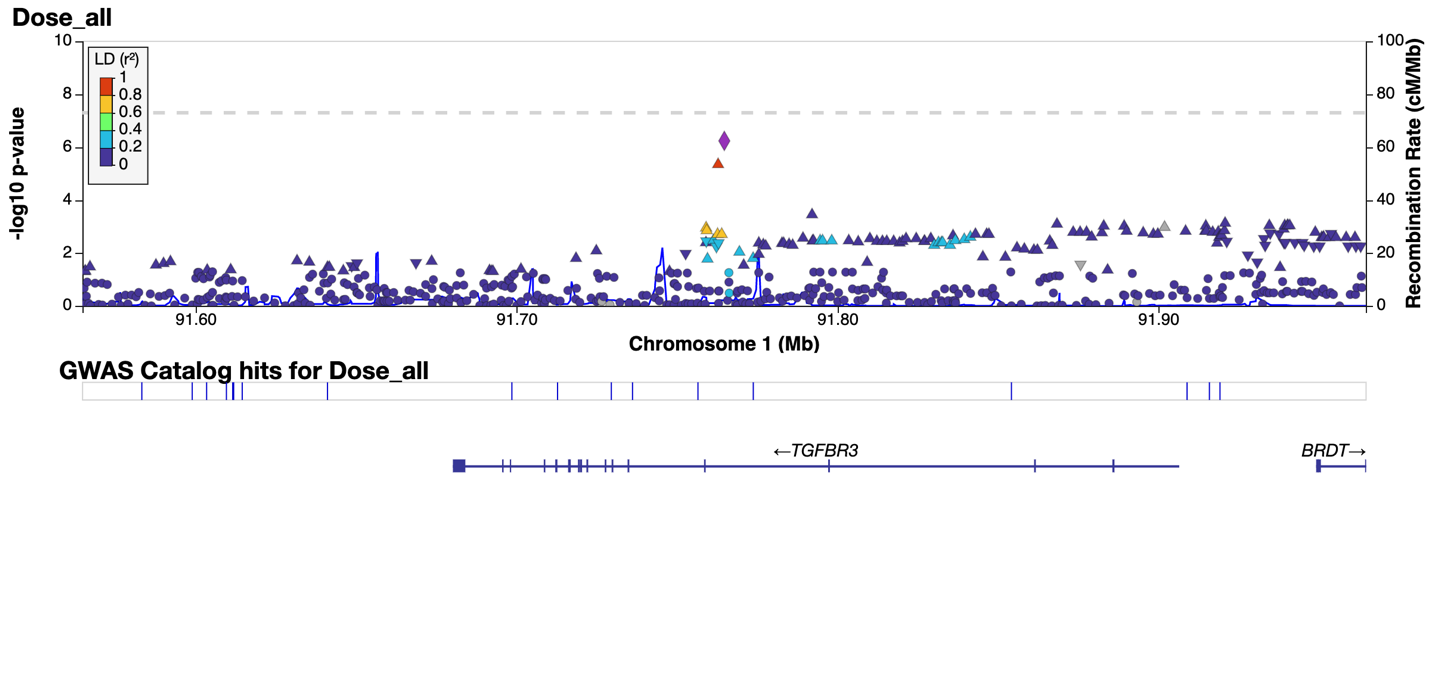


Supplementary Figure 10. Opioid overdose Manhattan Plot (n=1,327)


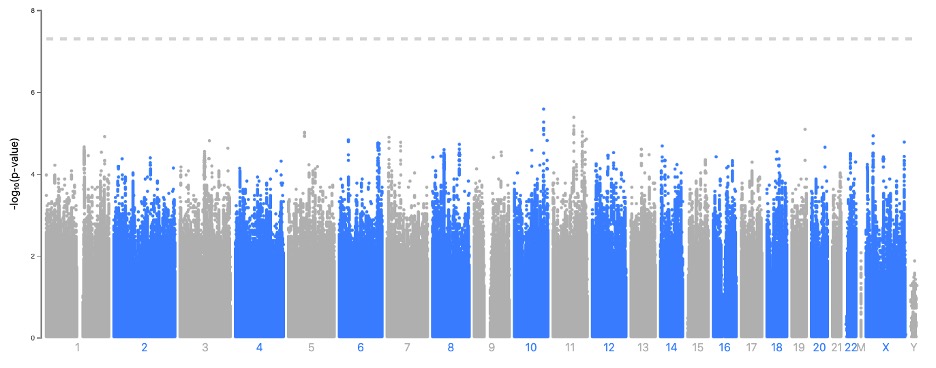


Supplementary Figure 11. Opioid overdose QQ Plot


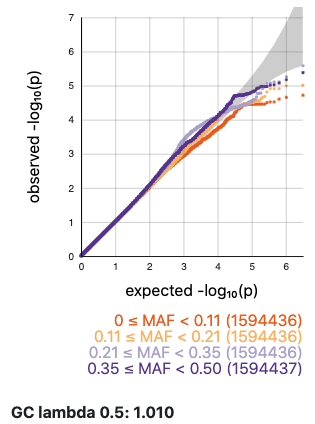


Supplementary Figure 12. Reginal plot for rs12777585 approaching genome-wide significance for opioid overdose from LocusZoom[1] using European for LD information


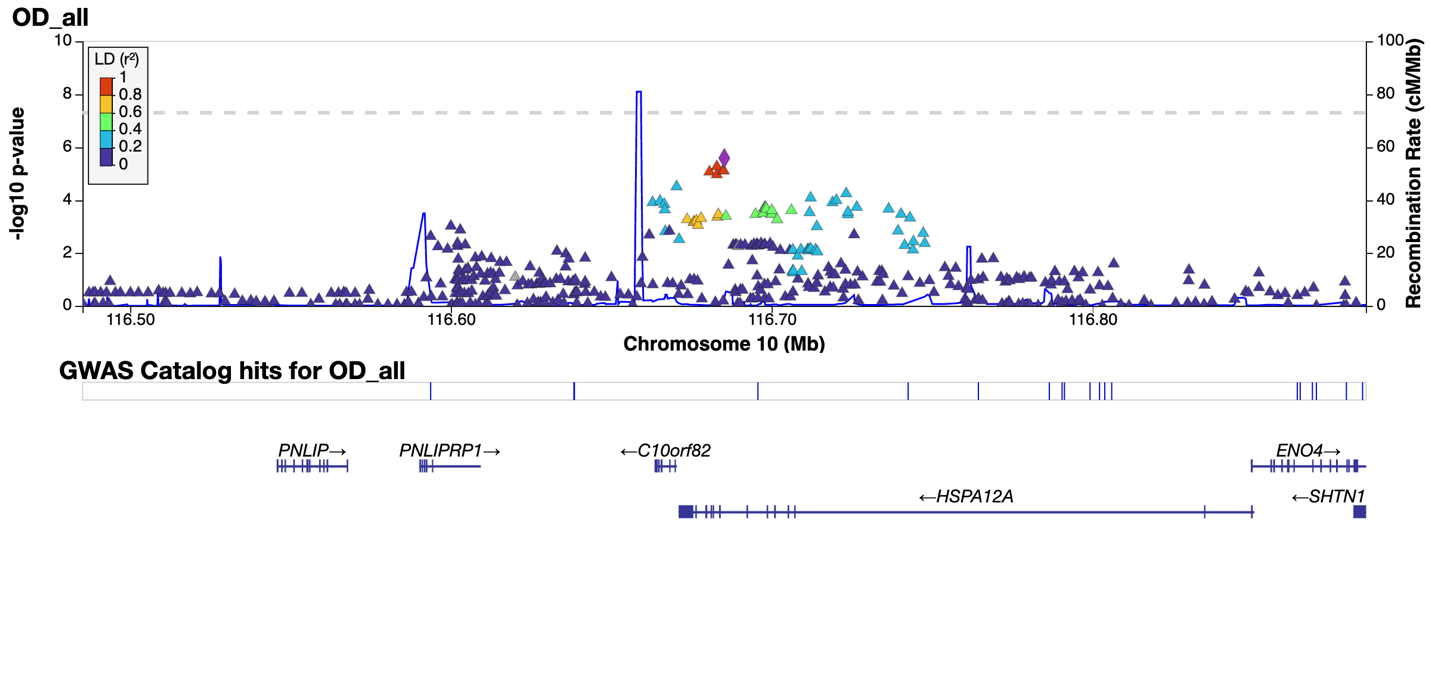


Supplementary Figure 13. Continued opioid use PRS model fit generated by PRcise[2]


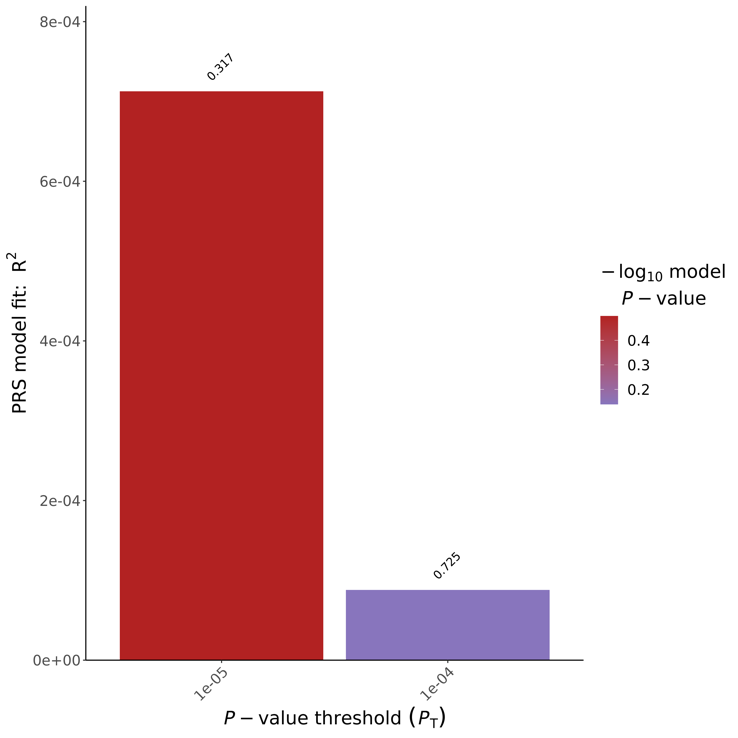


Supplementary Figure 14. Relapse PRS model fit generated by PRcise[2]


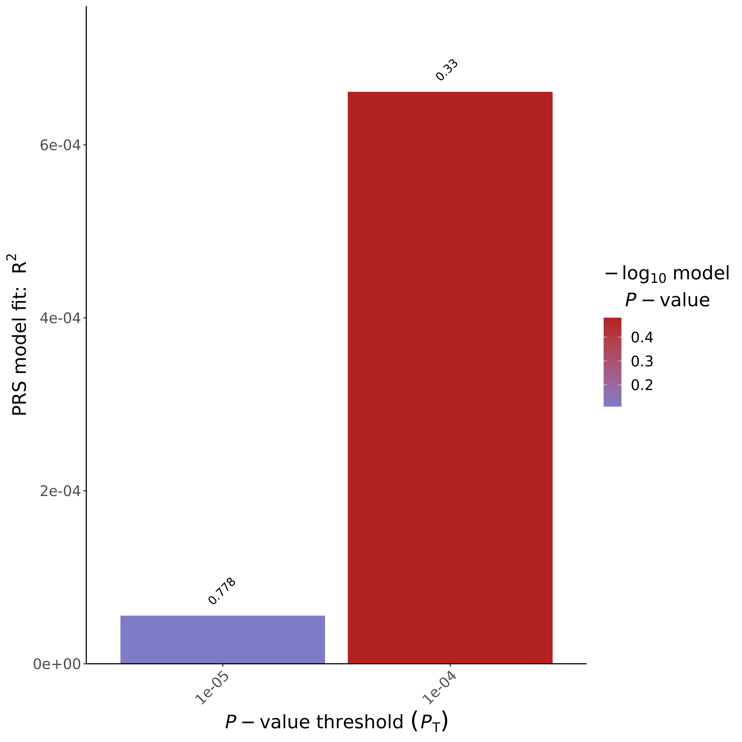


Supplementary Figure 15. Methadone dose PRS model fit generated by PRcise[2]


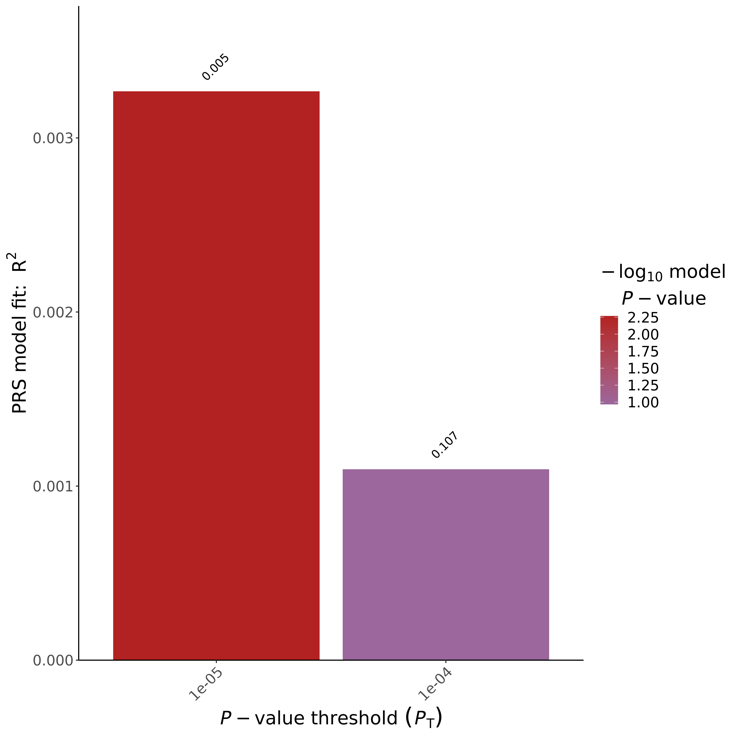


Supplementary Figure 16. Opioid overdose PRS model fit generated by PRcise[2]


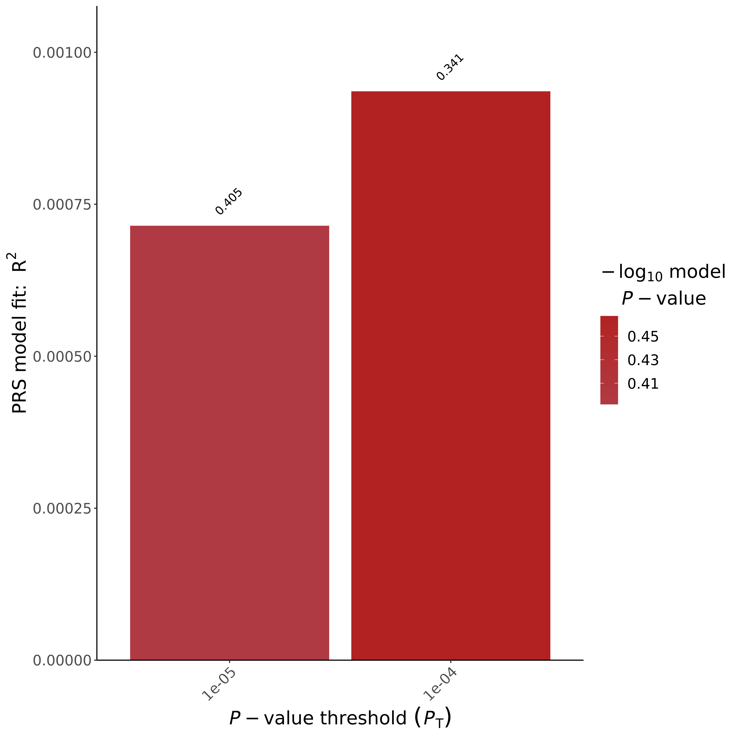


Supplementary Figure 17. LD plot for *HSPA12A* SNPs previously associated with externalizing behaviours, educational attainment, smoking initiation and rs12777585, associated with opioid overdose, generated by LDmatrix using a European population[3]


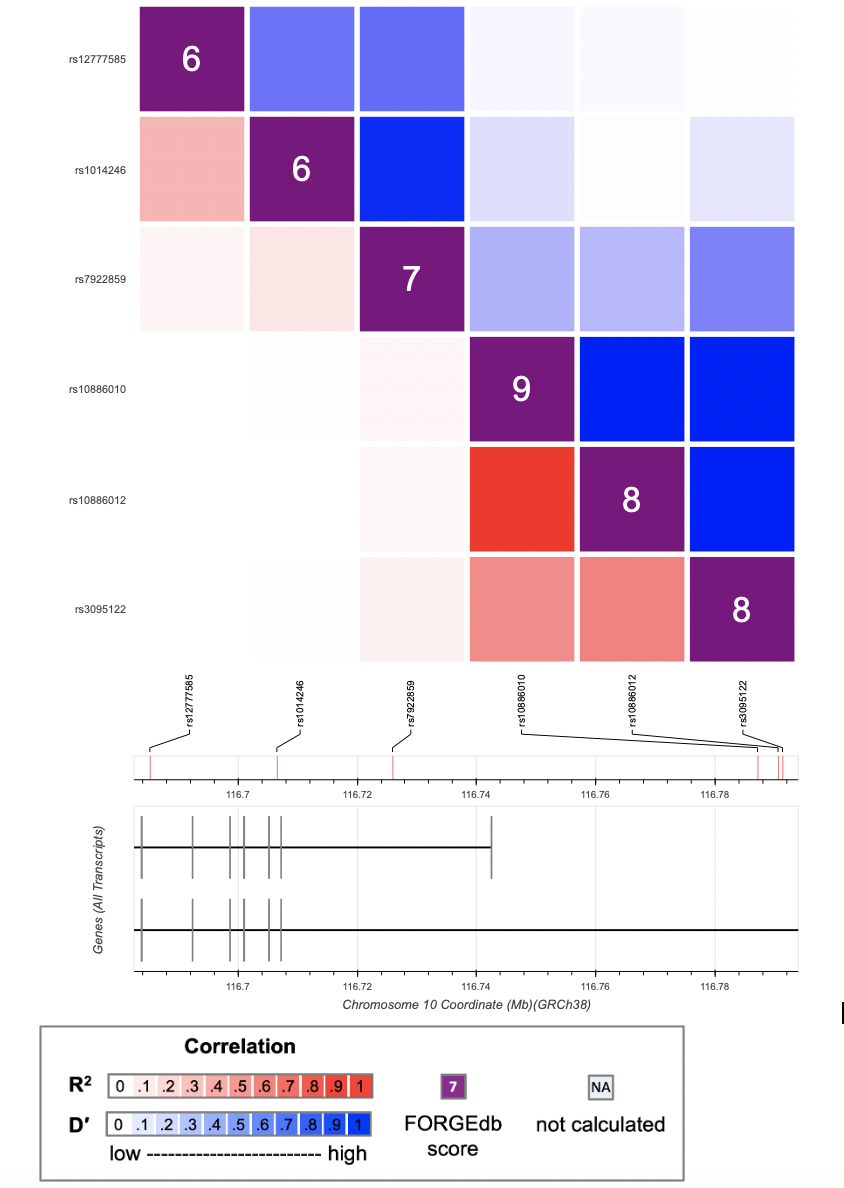


**References**

[1] A. P. Boughton *et al.*, “LocusZoom. js: Interactive and embeddable visualization of genetic association study results,” *Bioinformatics*, vol. 37, no. 18, pp. 3017–3018, 2021.

[2] S. W. Choi and P. F. O’Reilly, “PRSice-2: Polygenic Risk Score software for biobank-scale data,” *Gigascience*, vol. 8, no. 7, p. giz082, 2019.

[3] M. J. Machiela and S. J. Chanock, “LDlink: a web-based application for exploring population-specific haplotype structure and linking correlated alleles of possible functional variants,” *Bioinformatics*, vol. 31, no. 21, pp. 3555–3557, 2015.
